# Case Report: Glycogen Storage Disease Type Ia in a Chinese Child Treated With Growth Hormone

**DOI:** 10.3389/fped.2022.921323

**Published:** 2022-06-17

**Authors:** Shimin Wu, Shusen Guo, Lina Fu, Caiqi Du, Xiaoping Luo

**Affiliations:** Department of Pediatrics, Tongji Medical College, Tongji Hospital, Huazhong University of Science and Technology, Wuhan, China

**Keywords:** GSD Ia, growth retardation, growth hormone treatment, compound heterozygous variant, G6PC gene

## Abstract

**Background:**

Glycogen storage disease type Ia is a rare metabolic disorder that leads to excessive glycogen and fat accumulation in organs, characterized by hepatomegaly, hypoglycemia, lactic acidemia, hyperlipidemia, hyperuricemia, puberty delay, and growth retardation. Here, we report on a patient with glycogen storage disease type Ia treated with growth hormone.

**Case Presentation:**

A 10-year-old boy had growth retardation for 6 years, and was admitted to clarify the cause of his short stature. We found that his bone age was 5.5 years, significantly lower than his physical age, while his serum IGF-1 and IGFBP-3 were 23.30 and 1620.0 ng/mL, respectively, both lower than normal. His medical history revealed that he had suffered from steatohepatitis, hyperlipidemia, and hypoglycemia since he was 11 months of age. Whole exome sequencing (WES) showed compound heterozygous mutations in exons 2 and 5 of the glucose-6-phosphatase (G6PC) gene on chromosome 17: c.G248A (p.R83H) and c.G648T (p.L216L). The patient was finally diagnosed with GSD Ia. After growth hormone (GH) treatment and corn starch therapy for 14 months, his height significantly increased (by 13 cm). The serum IGF-1 level increased to the normal range but his lipid levels and liver function did not significantly increase.

**Conclusion:**

We describe a young patient with a compound heterozygous G6PC variant in a Chinese family; his height increased significantly after growth hormone and corn starch interventions. This case emphasizes that WES is essential for early diagnosis, and that growth hormone treatment may increase the height of patients with GSD Ia safely.

## Introduction

Glycogen storage disease type I (GSD I) is an autosomal recessive disorder resulting from insufficient activity of glucose-6-phosphatase (G6Pase), an enzyme that catalyzes the hydrolysis of glucose-6-phosphate (G6P) into glucose and inorganic phosphate, a key step in maintaining glucose homeostasis (1). There are two major GSD I subtypes: GSD type Ia [GSD1a (MIM232200)], which is the result of a mutation affecting the G6Pase-alpha (G6Pase-α or G6PC) catalytic subunit, and GSD type Ib (GSD Ib), which is caused by a defect in G6P

translocase (G6PT) (2). The incidence of GSD I is approximately 1 in 100,000–400,000 births, and GSD Ia accounts for 80%, making it the most common glycogen storage disease (3). GSD Ia mainly manifests as hepatomegaly, hypoglycemia, lactic acidemia, hyperlipidemia, hyperuricemia, delayed puberty, and growth retardation (3). These can be prevented by optimizing metabolic control of triglycerides, uric acid, lactate, and liver transaminases (4).

Growth retardation is one of the main symptoms of GSD Ia, but the underlying mechanism is unclear (1). The most probable cause is disruption of the growth hormone (GH)–insulin-like growth factor (IGF) I axis in patients with GSD Ia (5). Growth hormone can efficiently and safely treated children with short stature, including growth hormone deficiency (GHD), idiopathic short stature (ISS), and small for gestational age (SGA), as well as children with growth retardation related to long-term glucocorticosteroid therapy (6, 7). However, the safety and efficacy of GH in GSD Ia patients for managing the growth impairment remains controversial (1). There are also few reports on the use of GH in children with GSD Ia. Here, we reported a Chinese child with GSD Ia diagnosed by genetic testing and treated with GH and corn starch.

## Case Presentation

A 10.75-year-old boy was admitted to the Department of Pediatric Endocrinology, Tongji Hospital, for assessment of growth retardation in September 2020. His height had been below average since 2014. He had been born at full term, with normal measurements: Length 50 cm, weight 3.3 kg. He was normal in terms of teething, walking, speaking, and intelligence compared to age- and sex-matched children. His medical history revealed that he had suffered from steatohepatitis, hyperlipidemia, and hypoglycemia since he was 11 months of age. Pathologically, his liver cells evidenced fatty changes, and PAS staining suggested reduced glycogen deposition in hepatocytes. To reduce the aspartate aminotransferase (AST) and alanine aminotransferase (ALT) and protect liver function, glutathione tablets 0.1 g bid and bifendate 12.5 mg bid had been prescribed at 1 year of age, together with egg lecithin tablets 0.2 g tid (to reduce the hyperlipidemia).

**FIGURE 1:**
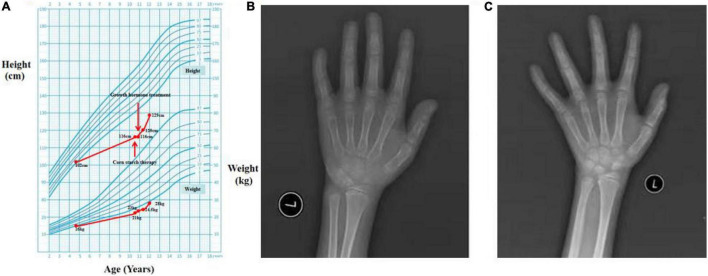
The growth curve and bone ages. **(A)** The growth curve of Chinese boys (8). **(B,C)** Plain X-rays of the left hand and wrist.

Physical examination revealed a short stature, a baby face, but normal limbs. His liver was palpable 2 cm below the belly-button line. He lacked red palms, telangiectases, and splenomegaly. He was 116 cm (SDS = −4.44) in height and 21 kg (< −2 SD) in weight, and his secondary sex characteristics were undeveloped (Tanner stage I, Testicular volume 1 ml) (Figure 1A) (8).

**FIGURE 2:**
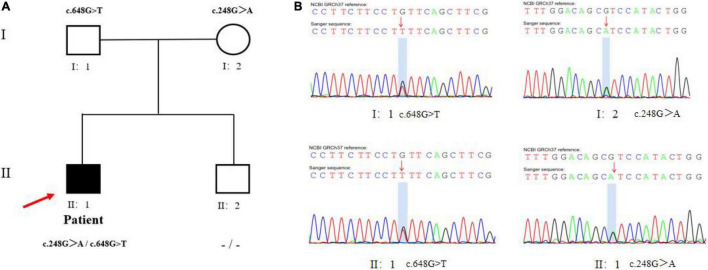
Mutational analysis in the patient pedigree. **(A)** The genotypes of G6PC gene for family members. Roman numerals indicate generations and Arabic numbers indicate individuals. Squares, males; circles, females. Affected individuals are denoted by solid symbols and unaffected individuals are denoted by open symbols. The index patient is indicated by an arrow. The two mutations were inherited from father and mother, respectively. **(B)** Validation for two compound heterozygous points mutations (c.G248A and c.G648T) at the exon 2 and 5 by Sanger Sequencing. The red arrow showed proband.

Laboratory tests revealed hypoglycemia, lactic acidemia, and severe dyslipidemia. In terms of the pituitary axis, the TSH and TT3 levels were normal. Spontaneous overnight GH profiling (12 h) revealed a mean GH level of 0.16 ng/mL; the IGF-1 and IGFBP-3 levels were both lower than normal at 23.30 and 1,620.0 ng/Ml, respectively, (Table 1). Hepatic ultrasonography revealed hepatomegaly and suspected GSD. On Tanner-Whitehouse (TW3) bone age assessment, his left-hand development score was 260, equaling the bone age of a 5.5-year-old male (Figure 1B), and was thus about 4 years behind that appropriate for his chronological age. Whole-exome sequencing revealed two compound heterozygous mutations in exons 2 and 5 of the G6PC gene (NM_000151) on chromosome 17: c.G248A (p.R83H) and c.G648T (p.L216L), which had been obtained from his mother and father, respectively. His younger brother was normal (no G6PC mutation). First-generation sequencing was performed to verify the mutations in the patient and his family, and the sequences were compared to the Human Gene Mutation Database^1^ (Figure 2).

**TABLE 1:** Examination data on presentation and follow-up.

Variables	Reference range	Time 1 (2010.11)	Time 2 (2011.03)	Time 3 (2014.08)	Time 4 (2018.10)	Baseline (2020.09)	2 months (2020.11)	5 months (2021.01)	8 months (2021.04)	11 months (2021.08)	14 months (2021.12)
Age (Y, M)*		11 M	1 Y 4 M	4 Y 9 M	8 Y 10 M	10 Y 9 M	10 Y 11 M	11 Y 2 M	11 Y 5 M	11 Y 8 M	12 Y
Height (cm)		-−	75	102		116	116	118	120	124.4	129
Weight (kg)		9	9	16		21	23	24.5	24.5	27	28
BMI						15.6	17.09	17.5	17.01	17.56	16.82
ALT (U/L)	4–41	138	62	247	162	159			136		148
AST (U/L)	4–41	148	253	189	128	167			280		161
Triglyceride (mmol/L)	0.05–1.70		9.56	7.42	9.88				14.24		6.59
Total cholesterol (mmol/L)	2.90–5.20	5.22	7.15	6.0	9.43	9.58			9.81		8.65
LDL-C (mmol/L)	0.03–3.12		4.95	3.65	3.59				5.55		
HDL-C (mmol/L)	1.10–1.90		0.63	0.82	1.35				0.65		
Uric acid (μmol/L)	202.3–416.5	443.8		598.5	628						
Creatine (μmol/l)	59–104	13		12	29.5						
Growth hormone (ng/ml)	0–10					0.78 ng/ml (0–10)					
IGF-1 (reference range)					<25 μg/L (64–345)	23.30 ng/ml (131 ± 47)		38.10 ng/ml (137 ± 54 ng/ml)	59.90 ng/ml (137 ± 54 ng/ml)		119.00 ng/ml (219 ± 78)
IGFPB-3 (reference range)					1.4 mg/L (1.6–6.6)	1620.0 ng/ml (3,244 ± 0)		3580.00 ng/ml (3,396 ± 678)	4400.00 ng/ml (3,396 ± 678)		5820.00 ng/ml (3,666 ± 750)
TT3 (nmol/L)	1.21–2.66					1.58		1.97	2.03	2.64	2.91
TSH (mIU/L)	0.6–4.5					5.62		5.36	5.03	7.20	2.84
Insulin (mIU/L)	0–18.95					1.98		1.0	3.9	7.52	5.87
Fasting blood glucose (mmol/L)	3.9–6.4	2.8				2.72		2.19	2.45	4.62	4.88
X-ray (Bone age)						5.5 years					7.0 years
Hepatic ultrasonography						Hepatomegaly, and fatty liver, suspected GSD.				Hepatomegaly, and fatty liver	
Gene report							c.G248A and c.G648T				
Growth hormone treatment							rhGH treament (0.2 μ/kg.d)	Continuously treatment	Continuously treatment	Continuously treatment	Continuously treatment
Corn starch therapy						Started taking (1.75 g/kg, 4 times a day)	Continuously taking	Continuously taking	Continuously taking	Continuously taking	Continuously taking

*M, months; Y, years.

We prescribed corn starch and a low-fat diet immediately after this patient was suspected of GSD Ia in September 2020. And 2 months later, this patient was diagnosed with GSD Ia and short stature, which were based on the clinical manifestations, auxiliary examinations, tissue pathology, and genetic testing. Because the patient’s height did not increase significantly after 2 months of corn starch therapy, we added growth hormone in November 2020. Since then, he has been followed-up regularly in the Department of Pediatric Endocrinology, Tongji Hospital. His data are shown in Table 1. When on recombination human growth hormone (rhGH) therapy with 0.2 U/kg⋅d and corn starch therapy (1.75 g/kg, 4 times a day), the patient showed a dramatic turnaround in growth (Figure 1A). After 14 months, his height increased 13 cm, a rate of about 2.6 cm every 3 months, and his bone age was 7 years (Figure 1C) at the age of 12 years. Both IGF-1 and IGFBP-3 had increased to the normal ranges. Moreover, the fasting blood glucose level improved and the incidence of hypoglycemia decreased during the treatment, and the latest fasting blood-glucose was 4.88 mmol/L, serum triglycerides 6.59 mmol/L, and total cholesterol 8.65 mmol/L (Table 1).

## Discussion

GSD Ia is an autosomal recessive, pan-ethnic disorder with genetic mutations identified in Asians, Caucasians, and Ashkenazi Jews (1). The presenting symptoms and signs vary with the patient’s age, and include hepatomegaly, hyperlipidemia, hypoglycemia, and poor growth (9). An early diagnosis and treatment help to decrease long-term complications in patients with GSD type Ia (4, 9). In this study, we present a case of GSD type Ia diagnosed by genetic testing; the patient’s height increased significantly after growth hormone intervention.

Currently, over 105 different mutations causing GSD Ia have been described in humans, and the typical metabolic disturbances are fairly consistent (10). The 2014 guidelines of the American College of Medical Genetics and Genomics recommended non-invasive molecular genetic testing to confirm GSD Ia when the clinical and laboratory evaluation suggest the diagnosis (1). Using non-invasive genetic testing from September 2002 to February 2019, we had detected 22 unreported gene variants in 49 Chinese patients with hepatic GSDs, including 3 novel variants of G6PC gene in 24 patients with GSD Ia (11). In this study, due to the lack of typical clinical symptoms, pathological features, and genetic testing, our patient was not diagnosed with GSD Ia when he presented with hepatomegaly and hyperlipidemia at the age of 11 months in 2010. When the patient was 10.75-years of age admitted to the Department of Pediatric Endocrinology, Tongji Hospital, we found heterozygous c.G248A (p.R83H) and c.G648T (p.L216L) mutations on WES, which have been reported in Chinese and South Koreans (10, 12). c.G648T is a well-known splice-site mutation and the most prevalent mutation (54% of the alleles), while p.R83H is present in 26% of the mutated alleles in Chinese patients (13). In addition, one Chinese patient with mild GSD Ia was missed early in life, but was later found to be a compound heterozygote (c.311A > T/c.648G > T) of G6PC (14). Ultimately, our patient was diagnosed with GSD Ia based on the clinical manifestations, laboratory results, and genetic testing result, which are essential for early diagnosis of the disease.

Growth retardation is one of the cardinal signs of GSD Ia, while there is no standard treatment due to unclear mechanism (1). Growth hormone has been used for over 35 years, and its safety and efficacy has been studied extensively in GDH, ISS, Turner syndrome, and intracranial and pituitary tumors (15). However, growth hormone in the treatment of severe growth retardation in children with GSD Ia is rarely reported. GH therapy had been reported to induce growth, but with variable responses in different patient groups with GSD Ia (5). One report stated that growth hormone injections were not effective in a boy with GSD Ia, who exhibited abnormal liver function and a hepatocellular carcinoma (14). Some researchers speculate that GSD Ia disrupts the hypothalamic–pituitary axis *via* an IGF-1 deficiency resulting from primary hepatic involvement (5, 16). Therefore, experts recommend that GH should be used carefully before the cause of growth retardation is identified (16, 17). At the beginning of corn starch therapy alone, the patient’s height did not increase significantly. When this was combined with growth hormone, the height improved dramatically (Table 1 and Figure 1A). Also, the fasting blood glucose level improved and the incidence of hypoglycemia decreased, which may have played important roles in promoting growth. We found that the IGF-1 and fasting growth hormone levels were below normal before GH replacement therapy. After combination treatment, the IGF-1 and IGFBP3 levels increased to the normal ranges, which may be related with GH therapy and stable blood glucose level in this patient. Moreover, We focused on the effects of growth hormone on the metabolism of lipids and liver function in the patient, and found that the serum TG and TC levels did not worsen, and AST and ALT improved. In summary, rhGH treatment significantly and safely increased the height of this patient at the 14-month follow-up (Table 1).

One shortcoming of this report is that no growth hormone stimulation tests were performed and related enzyme activities were not assessed. Additional studies are required to identify the pathogenic mechanisms of G6PC abnormalities in GSD Ia, which may be helpful to diagnose and treat patients. Moreover, the more number of cases and clinical data are important to confirm the efficacy and safety of growth hormone treatment in children with GSD Ia. Meanwhile, we will monitor the patient’s blood glucose, fatty liver, lactic acidemia, and hyperlipidemia, and check for hepatic adenomas in the long-term.

## Conclusion

We reported a Chinese child with GSD Ia with compound heterozygous point mutations (c.G248A and c.G648T) in exons 2 and 5 of the G6PC gene, whose height increased significantly after growth hormone intervention and corn starch therapy. Our work emphasizes that WES is essential for early diagnosis, and that growth hormone may increase the height of GSD Ia patients safely.

## Patient’s Perspective

The patient: “After having the daily injection of rhGH and corn starch therapy, I am so surprised that I can gain height at a speed that I had never imaged. However, the injection of rhGH is a little bit painful and troublesome, and I often have corn starch at 3 a.m. The doctor suggested me to not eat fat food and how to protect myself in daily life, which is important to defeat the disease.”

His mother: “I am very happy, my son has grown a lot taller. But treatment cost is increasing, and I will persist on the treatment. I am also worried about the inherited disease. Luckily, my son said that he felt well and the lab test results were roughly improved.”

## Data Availability Statement

The original contributions presented in this study are included in the article/supplementary material, further inquiries can be directed to the corresponding author.

## Ethics Statement

This study was approved by the Ethics Committee of Tongji Hospital (TJ-IRB20220117). Written informed consent to participate in this study was provided by the participants’ legal guardian/next of kin. Written informed consent was obtained from the minor(s)’ legal guardian/next of kin for the publication of any potentially identifiable images or data included in this article.

## Author Contributions

XL substantially contributed to the conceptualization of the manuscript. XL, SG, LF, and CD contributed to the acquisition and interpretation of the clinical data. SW and XL drafted and revised the manuscript. All authors read and approved the final manuscript.

## Conflict of Interest

The authors declare that the research was conducted in the absence of any commercial or financial relationships that could be construed as a potential conflict of interest.

## Publisher’s Note

All claims expressed in this article are solely those of the authors and do not necessarily represent those of their affiliated organizations, or those of the publisher, the editors and the reviewers. Any product that may be evaluated in this article, or claim that may be made by its manufacturer, is not guaranteed or endorsed by the publisher.

## Footnotes

1
www.hgmd.org
